# Evolution of the affective state of a cohort of people suffering from long covid and associated factors

**DOI:** 10.1192/j.eurpsy.2023.1643

**Published:** 2023-07-19

**Authors:** B. Oliván-Blázquez, S. León-Herrera, M. Samper-Pardo, F. Méndez-López, A. Aguilar-Latorre, M. Martinez-Perrochan

**Affiliations:** 1 University of Zaragoza; 2 Institute for Health Research Aragón, Zaragoza, Spain

## Abstract

**Introduction:**

Long COVID patients have experienced a decline in their quality of life caused, in part but not wholly, by its negative emotional impact. Some of the most prevalent mental symptoms presented by Long COVID patients are anxiety, depression and sleep disorders.

**Objectives:**

The objective of this study is to increase understanding of the affective state of people diagnosed with Long COVID, the evolution and associated factors.

**Methods:**

Longitudinal study of three months of duration. The study population was 100 post-COVID-19 patients aged 18 years or older (80 women and 20 men). The main variable was the affective state through the Hospital Anxiety and Depression Scale (HADS) questionnaire. The rest of the collected variables were: Socio-demographic variables, number of residual symptoms, cognitive functioning using the Montreal Cognitive Assessment (MoCA), physical functioning variable measured by Sit to Stand Test and Sleep quality through the Insomnia Severity Index (ISI). A statistical analysis comparing baseline and 3months follow up measures were performed, using a Student T for related samples statistical. A lineal regression analysing associated factors to a reduction in HADS score was also performed. Ethics approval was granted by the Clinical Research Ethics Committee of Aragón (PI21/139 and PI21/454).

**Results:**

At baseline the score in anxiety, depression and total score were 9,10 (SD: 4,67), 8,25 (SD: 4,51) and 17,35 (SD: 8,43) respectively, and 74% of the participants were considered cases. At three months, there is a slightly decrease but not significative in the score of HADS, both in anxiety, depression and total score (pvalue 0,465; 0,236; and 0,216 respectively). 64,4% of the participants had a positive diagnosis of depression/anxiety. About the rest of the variables there were also a slight decrease but without being significant There was not a predictive model that explained the decrease in the HADS score.

**Conclusions:**

The evolution of the people suffering long covid is very slow along the time, and also the affective state.

**Disclosure of Interest:**

None Declared

